# Liverpool Royal Infirmary

**Published:** 1891-10-10

**Authors:** 


### Liverpool Royal Infirmary.

By our Special Commissioner.

It is quite twenty years since we visited the Royal Infirmary, Liverpool. The old infirmary of those days was a remarkable institution, and competent critics gazed and wondered why it was not removed off the face of the earth. For instance, sanded floors and staircases were actually a boast of the institution. "So clean and tidy, you know," said our conductor of twenty years ago, "and all moisture is so speedily soaked up by the sand." It seems almost impossible to realise now that a hospital for the treatment of disease could ever have been quite what the old Royal Infirmary was. The nursing was also in a developing condition, but the period of development reached at the time we write of did not include intelligent and educated women as nurses, nor the enforcement of strict discipline and efficient management throughout the buildings. The condition of much which a visitor found existing in the ward kitchen and in connection with the use of apparatus was so disgraceful as to be past belief. We cannot even reproduce now the use to which enemata, for instance, were sometimes put by the nurses of those days, or bluntly state the kind of drippings which were at times allowed to drain into the beef-tea tins. All this is matter of history, and may therefore be relegated to the category of forgotten things except only that a restatement or a hint may be useful as revealing the enormous developments which have taken place in twenty years in hospital affairs in Liverpool, and, of course, throughout the country.

But what of the Royal Infirmary, Liverpool, of 1891? Built from plans by Mr. Waterhouse, R.A., the elevation and internal appearances are bound to be good. Of the general plan and some of its details points might readily be found to criticise and condemn, seeing that it is the first Institution Mr. Waterhouse has built; but, thanks to the exceptional ability and great practical thoroughness of Miss Staines, the Lady Superintendent, nothing has been allowed to be done without a struggle which could in any sense seriously militate against the general efficiency of the buildings for hospital purposes. How much Mr. Waterhouse and the Infirmary Committee owe to Miss Staines, and to what extent the architect has been saved from a multiplicity of small defects by the practical wisdom of the Lady Superintendent, it is not for us to precisely state. Let it suffice that the result justifies the partnership in the present instance, and shows how much it is to the advantage of an architect, nay how necessary it is, that he should have the aid of some one of the trained experience and knowledge of Miss Staines if he is to produce a hospital building which in practice will prove satisfactory and adequate to the medical and hygienic requirements of the day. Speaking broadly, the Royal Infirmary building comes out remarkably well; its arched corridors, its circular wards, its tiled staircases and chapel, and the remarkable completeness of the kitchen and offices, placed as they are at the top of the buildings, all deserve commendation.

We were much struck with the practical common sense displayed in various appliances in use at the hospital. In the nursing department we especially noticed the dressing trollies for the daily removal of used dressings from the wards, the Rufford's slop sinks, of which two appertain to each ward, supplied separately with hot and cold water, and which are provided with pedals so that the water can be turned on and off with the foot, whilst the hands are engaged. Dressing trays of Silesian enamelled ware have been especially made for the hospital. For the patients, bed tables, each with a table-cloth, are provided, and remarkably complete bedside cupboards. Meals are despatched to the ward kitchen in admirably arranged trollies, in which are separate divisions for meat, pudding, beef-tea, &c., with hot water heating apparatus. In the wardmaids' cupboard, above each pail, is a fixed wire cage, in which the scrubbing brush and flannel is kept—a very practical arrangement.

When to these features we add the marked efficiency of the administration of the hospital from ceiling to cellar, as shown by the condition of the wards and offices, the quietness and comfort of everything within the hospital proper, the touching thoughtfulness of the arrangements in the mortuary, which all ought to see and be grateful for, and the brightness and happiness of the staff in their work, their institution, and their officers, we may well congratulate the people of Liverpool upon the possession of so complete a hospital, and so excellent and practical a staff. Of the latter it is not too much to say that they make one wish that every great English hospital was equally fortunate in this particular respect.

It is pleasant to accord praise where praise is due, but in justice we are bound to regard all sides of the picture. One startling defect is apparent, and one that needs prompt remedy. We allude to the servants' cubicles, which are so unsatisfactory as to make it impossible to understand how Mr. Waterhouse could allow his name to be associated with anything so bad in a new building upon which a large sum of money has been expended. It is due to himself and his profession that he should re-model the servants' sleeping quarters (the day quarters are excellent), and it would be a graceful act, having regard to the present unsatisfactory accommodation, that he should execute the work at his own expense.

